# How *PLOS Biology* aims to foster diversity, equity and inclusion in science

**DOI:** 10.1371/journal.pbio.3001102

**Published:** 2021-03-09

**Authors:** The PLOS Biology Staff Editors

**Affiliations:** Public Library of Science, San Francisco, California, United States of America and Cambridge, United Kingdom

## Abstract

A diverse scientific community is not only fairer but also improves science overall by bringing a richer range of perspectives to the research enterprise. Here, we discuss steps that *PLOS Biology* is taking to support diversity, equity and inclusion at the journal and beyond.

There was a moment of reckoning last year after the release of a shocking video documenting the death of George Floyd, an unarmed Black man, at the hands of a white United States police officer. In response, people of conscience across the US and around the world took to the streets to demand an end to the systemic racism embedded in social institutions that perpetuate disparities from healthcare and housing to education and jobs—including those in science and medicine. The social movement that ensued, largely led by Black Lives Matter, also elicited reflection and acknowledgement of the racism and profound inequalities permeating the scientific enterprise. Scientific journals reflect the society they operate in—it’s thus no surprise that lack of access to opportunities has led to underrepresentation of women and ethnic minority scientists, from authors and peer reviewers to editors and publishers.

Several bioscience and medical journals, including PLOS, grappled with this question through editorials, blogs, and commentaries acknowledging structural racism and other inequalities and pledging to do better. Journals certainly have a part to play in fostering diversity and inclusion, but good intentions aren’t enough. As the New York Times noted in October, publishers for the most part lack diversity data on their contributors and reviewers or are only beginning to track it. The process for tracking diversity is not straightforward because privacy regulations, which differ between countries, can affect how race, ethnicity, and gender data are collected and stored. Yet journals can’t reliably increase diversity, equity, and inclusion without establishing a baseline to track progress. That’s why PLOS is committed to assessing the existing diversity of the contributors to our family of journals, starting with our Academic Editors but ultimately also including authors and reviewers. Having a benchmark measure will enable us to hold ourselves to account and monitor progress toward the goal of increasing the diversity of PLOS journals and their leadership. *PLOS Biology*, for example, has 8 professional editors, almost all of whom are white, and we recognize the limitations this inherently poses to our range of perspectives. In 2019, we made a targeted attempt to increase the female presence on the *PLOS Biology* editorial board, which currently stands at 42%, a percentage we plan to increase this year. We also aim to increase the board’s geographic, racial, and ethnic diversity, as well as recruit more researchers at earlier stages of their career. We are actively working to ensure a diversity of voices in our commissioned content and in our reviewer pool and routinely ask declining reviewers to keep diversity in mind when suggesting alternatives.

Science’s diversity problem doesn’t end with race and ethnicity. Transformative social change requires dismantling the biases and barriers that place all marginalized people and communities across the human experience at a disadvantage, from BIPOC to LGBTQ+. Improving diversity and inclusion requires addressing the needs of all research communities, which implies listening and understanding the obstacles they face. PLOS recently responded to concerns raised by transgender and nonbinary authors around changing their publishing record from the name they were given at birth, often referred to as a dead name, to their chosen name. In October, PLOS announced a new name change policy across the portfolio to honor those requests in order to prevent stigmatization and ensure that “the name on the paper reflects who authors are.” Changes to authorship in published articles are usually associated with the publication of a Correction Notice, but to protect the privacy of these authors and prevent further discrimination, no notice will be attached. The relevant articles will be “republished,” meaning that they will be fully replaced online and that their indexing metadata (which affects how the author list appears in PubMed, Web of Science, Google Scholar, etc.) should subsequently be updated accordingly. This replaces the author name fully while ensuring that citation information, such as the DOI, remains the same, and all previous citations to the paper remain valid.

*PLOS Biology* vows to offer a platform to shed light on inequalities, racism, and other discrimination that minorities and/or marginalized populations grapple with. Toward that end, this issue features a Perspective by Leo Chan Gaskins and Craig McClain proposing a novel solution for transgender authors to change their published names [1] and an Essay by Robinson Fulweiler, Sarah Davies, and other scientist mothers from different backgrounds and career stages that outlines how the Coronavirus disease 2019 (COVID-19) has exacerbated the inequalities that mothers in academia have long endured [2]. As Gaskins and McClain make clear, even when publishers like PLOS commit to updating authors’ names, the reality of approaching every publisher, which may or may not honor the request, places a disproportionate burden on transgender researchers. Invisible name changes, ideally propagated automatically through a centralized mechanism, would be a matter of dignity for trans researchers, they argue. “This would prevent their own publication record from outing them without their consent. A single, centralized name change request through ORCID iD would alleviate the burden of changing each publication individually.” Fulweiler, Davies, and colleagues highlight that “women scientists who are parenting while also engaging in a combination of academic-related duties are falling behind.” What’s more, the pandemic has created additional disparities for mothers of color. They recommend strategies that will ultimately benefit many groups by breaking free from old norms and could redress the problems that mothers in science face well beyond the current pandemic. “Rather than rebuilding what we once knew,” they write, “let us be the architects of a new world.”

Inclusivity in publishing also requires addressing the existing economic hurdles to publication and access to the published literature. To make selective open access more inclusive, equitable, and sustainable, *PLOS Biology* and *PLOS Medicine* are pioneering a collective action publishing model, PLOS Community Action Publishing (CAP). CAP is designed to protect authors from the rising costs of selectivity by equitably shifting publishing costs from authors to the institutions of both corresponding and contributing authors, and redistributing any revenue beyond the target back to community members. All Research4Life countries will automatically be members of the collective at no cost to them, so researchers in those countries will never be subject to fees, and authors based elsewhere and unable to pay nonmember fees can continue to apply to the fee waiver programs offered by PLOS. With this model, PLOS aims to ultimately completely eliminate author fees.

Clearly, scientific journals cannot single-handedly reshape the deeply embedded social forces that perpetuate racism, discrimination, and inequality in science. But they can monitor the effects of lack of diversity in the scientific community and ensure that they don’t perpetuate inequities by actively reducing the overwhelming homogeneity of their staffs, editorial boards, authors, and reviewers. They can expose the many challenges marginalized groups continue to face and showcase recommendations and policies that lay out paths to meaningful change. Many scientists entered their fields with the hope of understanding the way the world works to make it a better place. It’s time for academics to leave the comfort of the ivory tower, recognize that the same conditions that helped them get there are keeping others out, and join efforts to open the door to academic advancement for all.
